# Supplementary material for: Prenatal stress perturbs fetal iron homeostasis in a sex specific manner
Source: Sci Rep. 2022 Jun 4;12:9341. doi: 10.1038/s41598-022-13633-z (PMC9167276; doi:10.1038/s41598-022-13633-z)
Supplement: Supplementary file 1 — Supplementary Information. [file 41598_2022_13633_MOESM1_ESM.docx]

**Supplementary Online Content**

Zimmermann P, et al., Prenatal stress perturbs fetal iron homeostasis in a sex specific manner.

**Table S1. Study outcome parameters**

**Table S2. Iron parameters**

**Table S3. Sex-specific linear regression of FSI and serum iron parameters**

**Table S4. Study outcome parameters for minimum adjustment sets: effect on serum ferritin and role of sex**

**Fig S1. Sex-dependent group difference in cord blood serum transferrin saturation levels**

**Fig S2. Machine learning feature importance ranking contributing to classification of stressed group and control group participants**

**Supplement N1. Additional information about causal inference analysis and causal diagrams**

**Table S1. Study outcome parameters**

| **Characteristics** | | **CG** | **SG** |  |
| --- | --- | --- | --- | --- |
|  |  | n=85 | n=79 | p |
| **Baseline** | |  |  |  |
|  | Gestational age at screening [weeks] | 34.0 (33.3–35.0) | 34.0 (32.6–34.9) | 0.304 |
|  | Gestational age at inclusion [weeks] | 36.7 (35.2–37.6) | 36.4 (35.3–37.4) | 0.612 |
|  | Age mother at study entry [years] | 33.4 (±3.7) | 32.7 (±5.1) | 0.307 |
|  | BMI at study entry [kg/m²] | 26.3 (24.4–28.9) | 27.8 (25.3–34.6) | **0.010** |
|  | BMI pregestational [kg/m²] | 21.5 (20.2–23.5) | 23.3 (20.7–27.5) | **0.013** |
|  | Score PSS | 9 (6–12) | 22 (20–24) | **<0.001** |
|  | Cortisol in maternal hair [pg/mg] | 88 (40–133) | 97 (61–165) | 0.104 |
|  | European/Caucasian | 78 (92) | 73 (92) | 0.879 |
|  | Married | 67 (80) | 55 (70) | 0.136 |
|  | University degree | 65 (77) | 46 (58) | **0.013** |
|  | Household income> 5000€/month | 49 (58) | 28 (35) | **0.004** |
|  | Smoking | 1 (1) | 7 (9) | **0.022** |
|  | Multiparity | 37 (44) | 38 (48) | 0.557 |
|  | Planned pregnancy | 75 (93) | 53 (67) | **0.001** |
|  | IVF / ICSI | 9 (11) | 2 (3) | **0.039** |
|  | Gestational diabetes | 2 (2) | 12 (15) | **0.003** |
|  | Autoimmune disease | 6 (7) | 13 (16) | 0.060 |
|  | Working status at screening | 3 (4) | 4 (5) | 0.502 |
|  | Iron supplement  FSI* | 31 (36)  –0.01 ((–0.36)–0.34) | 35 (44)  0.38 ((–0.22)–0.75) | 0.307  **0.024** |
| **Perinatal outcome** | |  |  |  |
|  | Gestational age at birth [weeks] | 39.9 (39.0–40.6) | 39.5 (38.6–40.6) | 0.148 |
|  | Birthweight [g] | 3526.9 (395.1) | 3484.0 (463.0) | 0.526 |
|  | Birthweight percentile [%] | 49.0 (28.3–71.8) | 55.0 (28.0–74.3) | 0.863 |
|  | Length [cm] | 52.9 (±2.5) | 52.8 (±2.6) | 0.919 |
|  | Head circumference [cm] | 35 (34–36) | 35 (34–36) | 0.412 |
|  | Cesarean delivery | 17 (20) | 27 (35) | **0.035** |
|  | Labor induction | 15 (18) | 19 (24) | 0.310 |
|  | Gender female | 41 (48) | 30 (38) | 0.137 |
|  | 5-min Apgar<7 | 3 (4) | 2 (3) | 0.691 |
|  | Admission to NICU | 3 (4) | 3 (4) | 0.912 |
| **Arterial plasma cord blood analysis results** | |  |  |  |
|  | Base Excess [mmol/L]  (n=78 CG, n=72 SG) | –5.5 (±3.3) | –5.2 (±3.0) | 0.557 |
|  | Lactate [mmol/L]  (n=53 CG, n=50 SG) | 4.4 (3.0–5.3) | 3.8 (3.0–4.8) | 0.317 |
|  | Glucose [mg/dL]  (n=57 CG, n=51 SG) | 84.0 (64.0–98.0) | 71.0 (63.5–91.5) | 0.338 |
|  | pH  (n=81 CG, n=77 SG) | 7.26 (±0.09) | 7.28 (±0.08) | 0.203 |
|  | PO2 [mmHg]  (n=66 CG, n=57 SG) | 21.1 (16.7–26.6) | 18.4 (13.6–23.5) | 0.102 |
|  | PCO2 [mmHg] | 50.8 (±10.2) | 49.4 (±9.2) | 0.382 |
|  | (n=69 CG, n=64 SG)  Leukocytes [G/L]  (n=53 CG, n=49 SG)  Neutrophils [%]  (n=53 CG, n=48 SG) | 14.6 (11.9–17.4)  51.0 (46.5–56.0) | 13.3 (10.2–17.6)  54.0 (47.0–61.0) | 0.291  0.249 |
| Data are mean (SD) using t-test, median (interquartile range) using Mann-Whitney U test or n (%) using Pearson’s Chi-squared test. Sample size is indicated as applicable. Differences with p-value < 0.05 are in bold.  PSS: Perceived stress scale; PDQ: Prenatal distress questionnaire; BMI: Body-mass index; NICU: Neonatal intensive care unit; ICSI: Intracytoplasmic sperm injection; IVF: In-vitro-fertilization | | | | |

*missing values for 11 CG and 14 SG

**Table S2. Iron parameters**

| Characteristics | CG  n=54 | SG  n=53 | p |
| --- | --- | --- | --- |
|  |  |  |  |
| Cord blood serum iron [μg/dL] | 151.5 (±37.3) | 141.4 (±38.5) | 0.172 |
| Cord blood serum transferrin [mg/dL]  Cord blood serum transferrin saturation [%] | 176.3 (162.2–205.9)  59.5 (±17.6) | 186.6 (165.8–217.0)  54.8 (±19.3) | 0.348  0.189 |
| Cord blood serum ferritin [μg/L]* | 242.4 (140.6–329.6) | 176.0 (106.4–267.0) | 0.134 |
| Cord blood serum hepcidin [ng/dL]  Cord blood plasma hemoglobin [mg/dL]**  Cord blood plasma MCV [fL]**  Cord blood plasma MCH [pg]** | 23.6 (13.4–39.24)  15.6 (±1.6)  104 (101–106)  35 (34–35) | 18.9 (9.2–36.9)  15.7 (±1.6)  104 (100–107)  35 (34–35) | 0.184  0.832  0.734  0.605 |
|  | n=74 | n=71 | p |
| Maternal prenatal plasma hemoglobin [mg/dL]  Maternal prenatal plasma MCV [fL]  Maternal prenatal plasma MCH [pg]  Maternal prenatal anemia: Hb<11mg/dL  Maternal postnatal plasma hemoglobin [mg/dL]***  Maternal postnatal plasma MCV [fL]***  Maternal postnatal plasma MCH [pg]*** | 12.3 (1.0)  87 (84–90)  30 (28–31)  8 (10.8)  11.1 (1.5)  87 (85–91)  30 (29–31) | 12.2 (1.1)  88 (83–90)  30 (28–31)  9 (12.7)  10.8 (1.3)  88 (85–91)  30 (28–31) | 0.376  0.766  0.862  0.537  0.164  0.669  0.683 |

Data are mean (SD) using t-test, median (interquartile range) using Mann-Whitney U test or n (%) using Pearson’s Chi-squared test.

*missing values for 1 SG

**missing values for 1 CG and 4 SG

***missing values for 3 SG

**Table S3. Sex-specific linear regression of FSI and serum iron parameters**

| Characteristics | Male newborns (n=58)  R² | p | Female newborns (n=49)  R² | p |
| --- | --- | --- | --- | --- |
|  |  |  |  |  |
| Iron [μg/dL] | 0.001 | 0.78 | <0.001 | 0.96 |
| Transferrin saturation [%] | 0.002 | 0.89 | <0.001 | 0.94 |
| Ferritin [μg/L]* | 0.003 | 0.96 | 0.048 | 0.13 |
| Hepcidin [ng/dL] | 0.028 | 0.23 | 0.004 | 0.84 |
|  |  |  |  |  |

Correlations were performed using Spearman’s rank correlation.

*missing values for 1 SG with male sex

**Table S4. Study outcome parameters for minimum adjustment sets: effect on serum ferritin and role of sex**

| Parameter | Exposure Group | Estimate of Ferritin (μg/L) | Robust Std. Error | 95% Confidence Limits | | Z | Pr > \|Z\| |
| --- | --- | --- | --- | --- | --- | --- | --- |
| **Adjustment for “Household income > 5000€/month” and “Maternal Age.''** | | | | | | | |
| POM | CG | 245.12 | 21.00 | 209.28 | 280.95 | 13.41 | <.0001 |
| POM | SG | 212.08 | 21.00 | 170.92 | 253.24 | 10.10 | <.0001 |
| Average Exposure Effect |  | –33.04 | 27.93 | –87.79 | 21.71 | –1.18 | 0.2369 |

**Adjustment for “University Degree” and “Maternal Age.”**

| POM | CG | 246.76 | 17.08 | 213.28 | 280.25 | 14.44 | <.0001 |
| --- | --- | --- | --- | --- | --- | --- | --- |
| POM | SG | 208.70 | 16.85 | 175.68 | 241.72 | 12.39 | <.0001 |
| Average Exposure Effect |  | –38.06 | 21.35 | –79.91 | 3.78 | –1.78 | **0.0746** |

**Adjustment for “Household income > 5000€/month”, “Maternal Age'' and “Fetus Sex.”**

| POM | CG | 245.15 | 19.45 | 207.03 | 283.28 | 12.60 | <.0001 |
| --- | --- | --- | --- | --- | --- | --- | --- |
| POM | SG | 212.48 | 16.44 | 180.27 | 244.69 | 12.93 | <.0001 |
| Average Exposure Effect |  | –32.68 | 24.31 | –80.32 | 14.96 | –1.34 | 0.1788 |

**Adjustment for “Household income > 5000€/month”, “University Education'' and “Fetus Sex.”**

| POM | CG | 248.07 | 20.48 | 207.93 | 288.21 | 12.11 | <.0001 |
| --- | --- | --- | --- | --- | --- | --- | --- |
| POM | SG | 213.01 | 20.61 | 172.60 | 253.41 | 10.33 | <.0001 |
| Average Exposure Effect |  | –35.06 | 26.73 | –87.44 | 17.33 | –1.31 | 0.1896 |

Differences in Average Exposure Effect with p-value < 0.1 are in bold.

POM: Potential Outcome Model


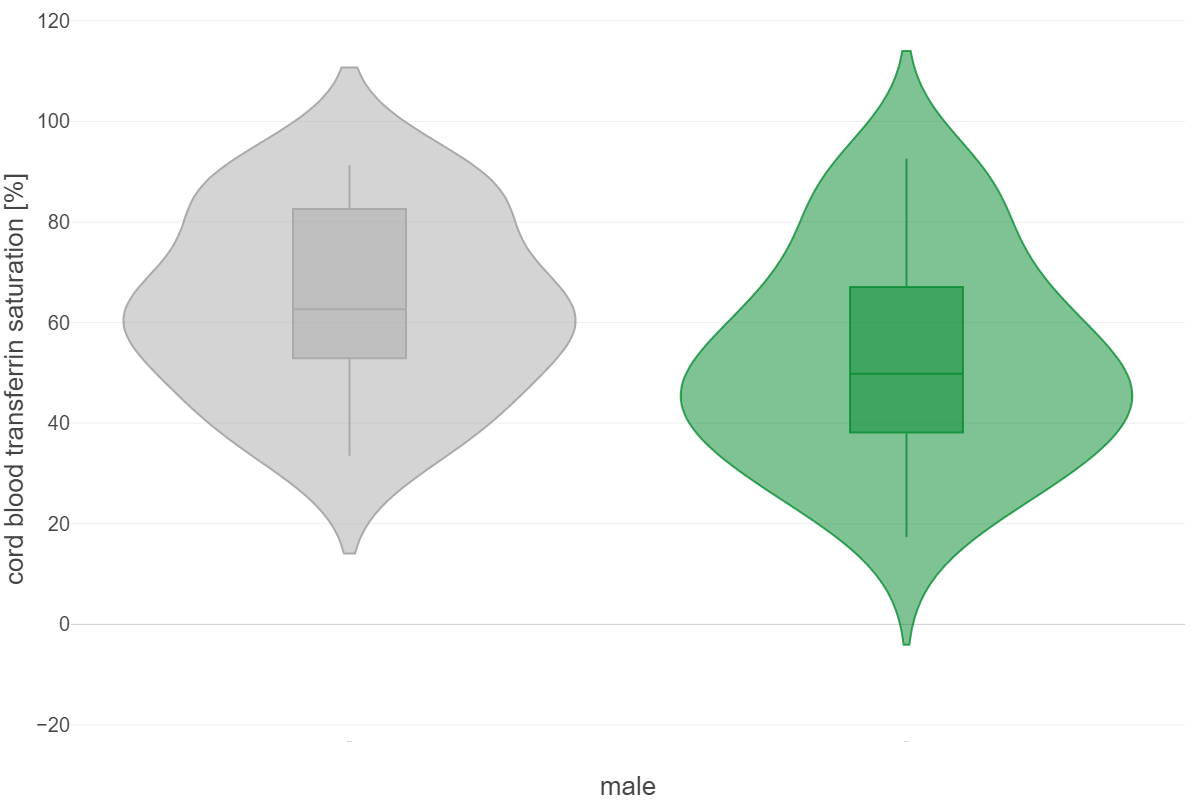


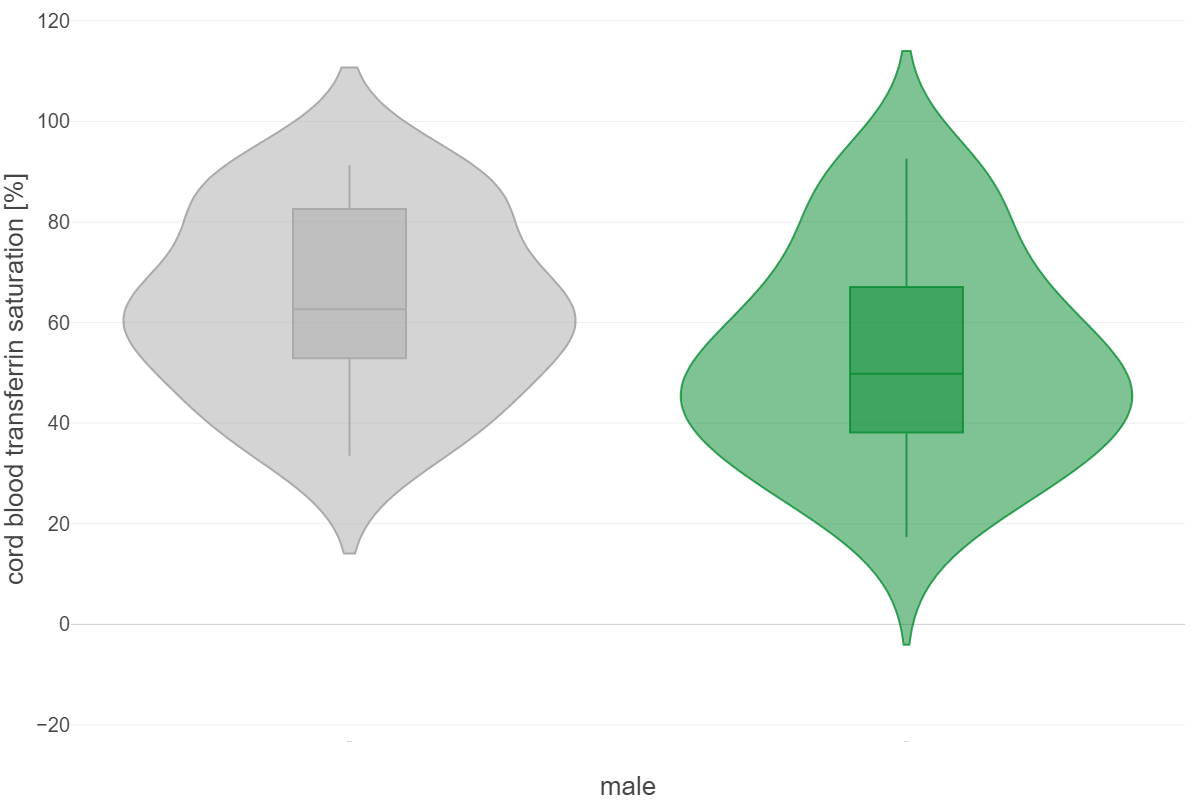

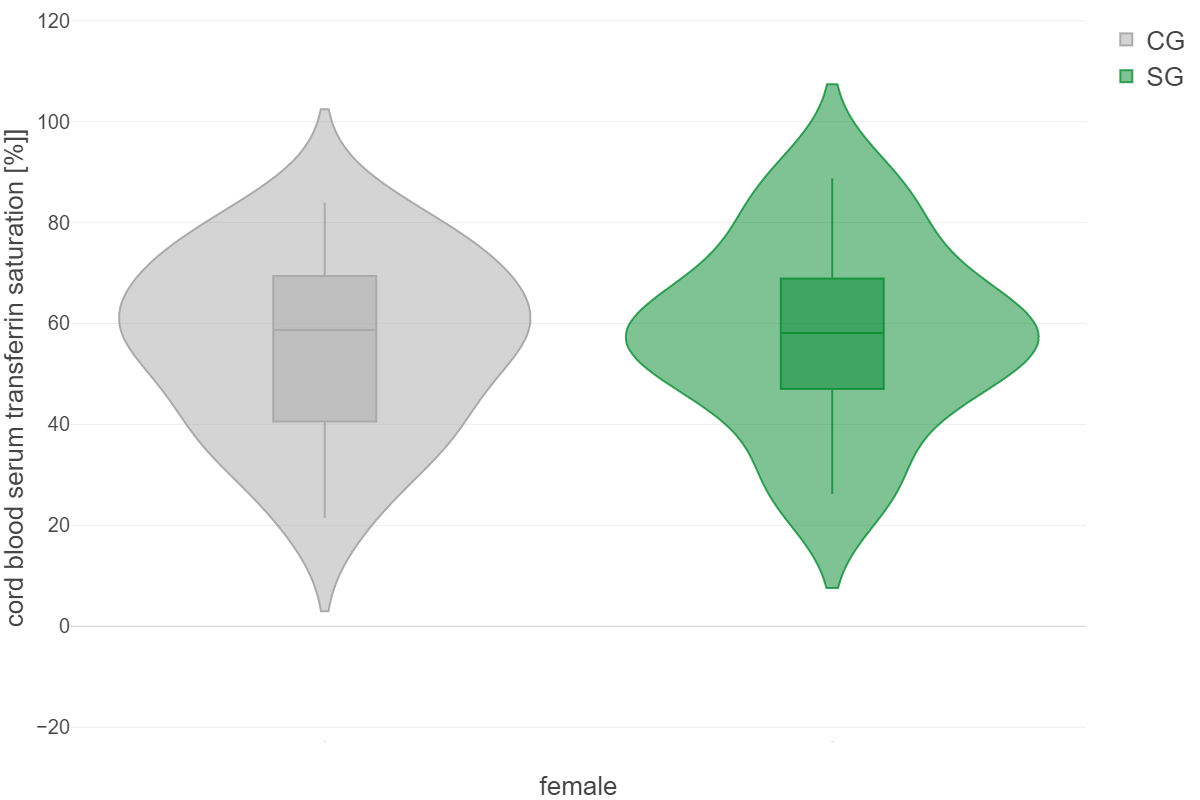


**Fig S1. Sex-dependent group difference in cord blood serum transferrin sat. levels**

GEE model the main effects of sex and study group and their interaction (sex*group) on transferrin sat. levels. GEE transferrin: group*sex p = 0.070

SG: Stressed group; CG: Control group; GEE: Generalized estimating equations

**Fig S2. Machine learning feature importance ranking contributing to classification of stressed group and control group participants**

FSI: Fetal stress index; BMI: Body-mass index

**Supplement N1. Additional information about causal inference analysis and causal diagrams**

The causal diagram shown in Fig. 3 was built as a conceptual model to demonstrate the authors’ assumptions about the factors influencing the neonates’ health outcome. As work by Greenland et al.^1^ has demonstrated, if these diagrams are constructed according to certain rules they can provide a rigorous way to address confounding. A correctly drawn causal diagram can be used to determine whether controlling for a certain combination of variation would be sufficient to remove confounding from the exposure-outcome association, or to identify variables that should not be controlled or that need not be controlled.

As this cohort study examined multiple maternal exposures, determining a causal diagram was necessary to aid in communication with our audience *and* to ensure that we controlled for any confounding, especially as confounding can also depend on what other variables have already been controlled for.

In terms of this study, we made use of the online software “dagitty”, a browser-based environment for creating, editing, and analyzing causal diagrams that makes use of the aforementioned causal diagram rules defined by Greenwood et al.

- An arrow denotes a direct causal effect: that is X→ Y implies that with all other variables held constant, changing X would change Y.
- While traditional approaches focus on individual variables, causal diagram theory focuses on how variables relate to causal paths and which set of control variables are sufficient to block those paths. In the case of Figure 3, pink arrows represent a direct causal effect along a biased path, while black arrows represent a direct causal effect along a non-biased path.
- The minimal adjustable set discussed in the manuscript is the minimum number of variables to ‘close’ all biasing paths (turn all pink lines to black) shown in the latter half of the new Fig. 3.

1 Greenland, S., Pearl, J. & Robins, J. M. Causal diagrams for epidemiologic research. Epidemiology 10, 37-48 (1999).
